# Prognosis-related molecular subtyping in head and neck squamous cell carcinoma patients based on glycolytic/cholesterogenic gene data

**DOI:** 10.1186/s12935-023-02880-3

**Published:** 2023-02-25

**Authors:** Zekun Zhou, Jianfei Tang, Yixuan Lu, Jia Jia, Tiao Luo, Kaixin Su, Xiaohan Dai, Haixia Zhang, Ousheng Liu

**Affiliations:** 1 Hunan Key Laboratory of Oral Health Research & Hunan 3D Printing Engineering Research Center of Oral Care & Hunan Clinical Research Center of Oral Major Diseases and Oral Health & Academician Workstation for Oral-maxilofacial and Regenerative Medicine & Xiangya Stomatological Hospital & Xiangya School of Stomatology, Central South University, Changsha, 410008 Hunan China; 2 The Oncology Department of Xiangya Second Hospital, Central South University, Changsha, 410011 Hunan China

**Keywords:** Bioinformatics_1_, Metabolic classification_2_, Prognosis_3_, Head and neck squamous cell carcinoma_4_, Glycolysis_5_, Cholesterogenic_6_

## Abstract

**Background:**

Head and neck squamous cell carcinoma (HNSCC) remains an unmet medical challenge. Metabolic reprogramming is a hallmark of diverse cancers, including HNSCC.

**Methods:**

We investigated the metabolic profile in HNSCC by using The Cancer Genome Atlas (TCGA) (n = 481) and Gene Expression Omnibus (GEO) (n = 97) databases. The metabolic stratification of HNSCC samples was identified by using unsupervised k-means clustering. We analyzed the correlations of the metabolic subtypes in HNSCC with featured genomic alterations and known HNSCC subtypes. We further validated the metabolism-related subtypes based on features of ENO1, PFKFB3, NSDHL and SQLE expression in HNSCC by Immunohistochemistry. In addition, genomic characteristics of tumor metabolism that varied among different cancer types were confirmed.

**Results:**

Based on the median expression of coexpressed cholesterogenic and glycolytic genes, HNSCC subtypes were identified, including glycolytic, cholesterogenic, quiescent and mixed subtypes. The quiescent subtype was associated with the longest survival and was distributed in stage I and G1 HNSCC. Mutation analysis of HNSCC genes indicated that TP53 has the highest mutation frequency. The CDKN2A mutation frequency has the most significant differences amongst these four subtypes. There is good overlap between our metabolic subtypes and the HNSCC subtype.

**Conclusion:**

The four metabolic subtypes were successfully determined in HNSCC. Compared to the quiescent subtype, glycolytic, cholesterogenic and mixed subtypes had significantly worse outcome, which might offer guidelines for developing a novel treatment strategy for HNSCC.

**Supplementary Information:**

The online version contains supplementary material available at 10.1186/s12935-023-02880-3.

## Introduction

Head and neck squamous cell carcinoma (HNSCC) is a heterogeneous disease comprising tumors of the oral cavity, lip, oropharynx, nasopharynx, larynx, hypopharynx and salivary gland [1]. HNSCC is the sixth most common malignancy in humans worldwide, with 930,000 newly diagnosed cases and 467,000 deaths in 2020 [2]. An estimated 1.37 million new cases are projected to occur in 2040, representing a 32% increase [3]. Current research indicates that HNSCC is closely related to numerous factors, including smoking, drinking, and human papilloma virus [1]. Despite advances in treatment strategies and improved prognosis, HNSCC remains an incurable malignancy, with approximately half of patients relapsing and dying from the disease [4]. Additionally, histopathology and clinical stage are not sufficient to accurately predict the prognosis of a patient because of the heterogeneity of HNSCC [1].

Recently, tumor-related metabolic reprogramming has been extensively studied, offering an approach to target cancers [5]. Metabolic reprogramming is recognized as a hallmark of cancer and presents opportunities for cancer diagnosis, prognosis, and therapy [6–8]. Cancer cells accumulate metabolic alterations to meet energetic demands and produce biosynthetic precursors, such as glucose, nucleotides, fatty acids and amino acids, for rapid tumor growth [8–11]. Such metabolic alterations can affect the fate of cancer. Recent studies have shown that the process of HNSCC emergence is related to tumor metabolism, which is mainly characterized by abnormal glycolysis and cholesterol synthesis. On the one hand, cancer cells utilize glycolysis for producing energy to promote the proliferation of cancer cells [12]. Molecular imaging studies using 18F-fluoro-2-deoxy-d-glucose positron emission tomography demonstrated increased glucose uptake and glycolysis in HNSCC [13, 14]. Increased glycolysis correlates with aggressive tumor progression, treatment resistance, and unfavorable prognosis in HNSCC [15, 16].On the other hand, cancer cells require high levels of cholesterol for membrane biogenesis and other functional needs, and subsequently promotes tumor development [17]. Meanwhile, certain investigations have indicated several key enzymes of cholesterol synthesis are closely related to poor prognosis of HNSCC [18]. Avasimibe, a specific inhibitor of ACAT, significantly inhibited tumor growth and prolonged survival by inhibiting the accumulation of cholesterol ester [19]. Therefore, it is worthwhile exploring prognostic significance base on glycolysis and cholesterol metabolism and the treatment underlying the metabolic perspective in HNSCC.

The difference of 2-DG uptake within patient HNSCC tumors raises the possibility that intertumoral differences in glycolysis [20] and the balance between glycolysis and cholesterol synthesis could regulate tumor aggressiveness [21]. HNSCC cell lines have distinct metabolic profiles which affect their response to metabolic agents [22]. However, whether heterogeneity in distinct metabolic profiles can be used to classify HNSCC into clinically relevant subtypes has not been well established. However, as the most recent guidelines indicated for the diagnosis of primary HNSCC, the pathology of HNSCC is mainly determined based on histological morphology. Compared with breast cancer, lung cancer, and gastric cancer [23–25], the molecular classification of HNSCC has fallen behind and cannot meet the needs for accurate clinical treatment. Therefore, it is important to understand energy metabolic reprogramming in HNSCC, which may offer a novel strategy for the further subtyping of HNSCC cases, thus facilitating the development of accurate and targeted therapy to improve the prognosis of patients.

In this work, we divided HNSCC cases into diverse subtypes according to the expression levels of genes related to cholesterol production and glycolysis. Consensus clustering analysis was applied to stratify 481 patients into four metabolic subtypes, and the stratification was further validated in the GEO cohort. This study examined the heterogeneities in survival as well as additional clinicopathological features across different HNSCC metabolic subtypes and detected carcinogenic molecular events among these diverse subtypes. Understanding the metabolic subtyping of HNSCC may therefore instruct the clinical management of this cancer and may help develop personalized therapies targeting metabolic pathways for prolonged patient survival.

## Materials and methods

### HNSCC dataset acquisition and processing

The GEO (https://www.icgc.org, GSE41613) [26] and TCGA (Illumina HiSeq Systems;) data portals were used to obtain HNSCC datasets together with related clinical data. In addition, the standard RNA sequencing data of the 481 TCGA-derived patients and 97 GSE41613-derived patients were collected. The human genome reference sequence GRCh37 formulated by the Genome Reference Consortium was used. In addition, somatic mutational data (SNVs, CNVs, and INDELs) were collected for each sample.

### RNA sequencing data analysis

RNA expression of every sample was normalized by the transcripts per million algorithms, which was later log-transformed into log10 ((normalized count*1e6145) + 1). A log2-fold change (FC) ≥ 1 was used as the threshold to select RNAs with significant differential expression. Samples whose tumor content was < 30% were eliminated from this work [27].

### Metabolic gene subgroup classification

Genes obtained from the gene sets of the molecular signatures database (mSigDB) [28], namely, “REACTOME CHOLESTEROL BIOSYNTHESIS” (n = 24) and “REACTOME GLYCOLYSIS” (n = 29), were identified as cholesterogenic and glycolytic genes, respectively. Then, these genes were subjected to consensus clustering by adopting ConsensusClusterPlus (parameters: reps = 100, pFeature = 1, pItem = 0.8) [29]. Meanwhile, the Euclidean distance (ED) and Ward. D2 were adopted as the distance matrix and the clustering algorithm, respectively, with k = 4 (Additional file 1: Figure S1). In addition, the median expression of coexpressed cholesterogenic and glycolytic genes was utilized to assign the quiescent (glycolytic ≤ 0, cholesterogenic ≤ 0), cholesterogenic (glycolytic ≤ 0, cholesterogenic > 0), glycolytic (glycolytic > 0, cholesterogenic ≤ 0), or mixed (glycolytic > 0, cholesterogenic > 0) metabolic subtypes for every sample.

### Pre-existing HNSCC subgroup classification

Samples were classified by consistent clustering according to common tumor subtypes investigated by Weidong Zhang et al.[30] and Hongbo Zhou et al.[31]. Typically, subtyping was classified according to 6 mRNA expression levels in the original paper by Weidong Zhang, whereas subtyping was categorized according to 3 mRNAs from the original study by Hongbo Zhou. In the classification process for every subtype, each sample was consistently clustered according to mRNAs in every classifier, followed by semiautomatic subtype assignment.

### Mutation analysis for HNSCC genes

Gene sequences were identified from the GRCh37/hg19 human genome assembly. To identify oncogenic events among diverse HNSCC metabolic subtypes at the molecular level, the frequencies of SNVs, CNVs and INDELs were detected from frequently mutated HNSCC genes [32], and their associations with diverse HNSCC metabolic subtypes were also explored. For tumor ploidy, we defined DNA fragments with copy statuses ≥ 3 as amplified, whereas those ≤ 1 were defined as deleted [33]. Additionally, HNSCC copy number events were selected according to prior work using 10 or more supporting probes at the threshold of mean fragment > 0.2 (amplified) or < -0.2 (deleted). Afterward, the copy number event coordinates were mapped into the gene coding region using maftools, whereas contingency analysis was applied to test CNVs and SNVs for every gene. Finally, we tested those genes screened from every subgroup.

### Pan-TCGA RNA-seq analysis

The RNA-seq data [RNA-seq by expectation maximization (RSEM); GRCh37] of each TCGA-derived non-HNSCC sample were obtained using the GDC data portal. Then, samples of different cancer types that had 100 or more samples were screened, and 262 cancer types were obtained. The expression levels were subjected to log transformation (log10(RSEMþ1)), and then genewise location scaling was used for batch correction in every cancer type. Typically, consensus clustering (ConsensusClusterPlus, parameters: pFeature = 1, pItem = 0.8, reps = 100; ED and Ward. D2, k = 4) was repeated for every individual cancer type based on gene expression in the “REACTOME CHOLESTEROL BIOSYNTHESIS” and “REACTOME GLYCOLYSIS” gene sets. Moreover, we determined the percentages of cholesterogenic and glycolytic genes in every cluster, and clusters consisting of at least 50% of each gene set were identified as the “core” clusters. With regard to cancer types that had over one core cluster in one gene set, we chose the most homogenous cluster as the core cluster. Cancer types without 75% or higher homogeneity in the core cholesterogenic and glycolytic clusters were eliminated from subsequent analysis, giving rise to 12 cancer types. Meanwhile, for every cancer type, we further determined its metabolic subtypes according to the median expression of representative core cholesterogenic and glycolytic genes.

### Survival of HNSCC cases

For this study the ‘‘survminer’’ v.0.4.2 and ‘‘survival’’ v.2.4.2 R packages were employed to generate Kaplan–Meier plots. Cases whose overall survival (OS) was shorter than 1 month were eliminated from survival analysis.

### Patient specimens and Immunohistochemistry (IHC)

A total of 22 tissue samples were obtained from patients with HNSCC, who underwent surgical resection at Xiangya Stomatological Hospital, Central South University between 2020 and 2022, including 12 recurrent HNSCC patient and 10 first diagnosed HNSCC patients surviving at least 5 years. All HNSCC patients were diagnosed by professional pathologist. All of 3 normal mucosa tissues were obtained during surgical removal of lower third molars and confirmed by pathologic examination. The study was approved by the Ethics Committee of Xiangya Stomatological Hospital of Central South University and informed consent was obtained from the patients. Clinicopathological characteristics with the patients were collected and presented in Additional file 1: Table S1. Paraformaldehyde-fixed tissue were embedded in paraffin and sectioned into 3-μm sections, deparaffinized, and rehydrated, followed by IHC protocol. Subsequently, 3-μm sections were incubated within 3% hydrogen peroxide for 15 min to block endogenous peroxidase activity and antigen retrieval was performed by microwave oven. Next, the slide were blocked in 5% goat serum for 15 min at 37 °C. Slides were incubated overnight at 4 °C with the following primary antibodies: anti-ENO1 rabbit polyclonal antibody (1:200; Proteintech, Wuhan, China), anti-PFKFBS rabbit polyclonal antibody (1:200; Proteintech, Wuhan, China), anti-SQLE rabbit polyclonal antibody (1:200; Proteintech, Wuhan, China), anti-NSDHL rabbit polyclonal antibody (1:200; Proteintech, Wuhan, China). Finally, slides were observed under a light microscope and evaluated based on the immunoreactive score (IRS) [34], which was multiplied by the staining proportion score and staining intensity score. The staining proportion was assigned a score of 0 (no staining), 1 (< 10% of positive cells), 2 (10–50% positive stained cells), 3 (51–80% positive stained cells) and 4 (> 80% positive stained cells). Staining intensity score was graded from 0 to 3 as follows: 0 = negative staining; 1 = weak staining; 2 = moderate staining; 3 = strong staining. According to IRS, patients were divided into three groups as follows: negative expression group, IRS = 0–1; mild expression group, IRS = 2–3; moderate positive expression group, IRS = 4–8; and strongly positive expression, IRS = 9–12. All slides were independently scored by two pathologists blinded to the clinicopathological features and outcome data.

### Statistical analysis

In this study, both GraphPad Prism 8 (San Diego, CA, USA) and SPSS 23.0 (IBM Corp., Armonk, NY, USA) were employed for data analysis. The levels of protein expression was analyzed by two-tailed Student’s t-test by comparing with the control group. Patient survival was analyzed by the log-rank test and Kaplan–Meier test. The optimal thresholds for every gene and every survival curve were obtained through R Studio. A difference of *P* < 0.05 (two-sided) indicated statistical significance.

## Results

### Identification and validation of HNSCC subtypes based on the expression levels of glycolytic and cholesterogenic genes

**Fig. 1:**
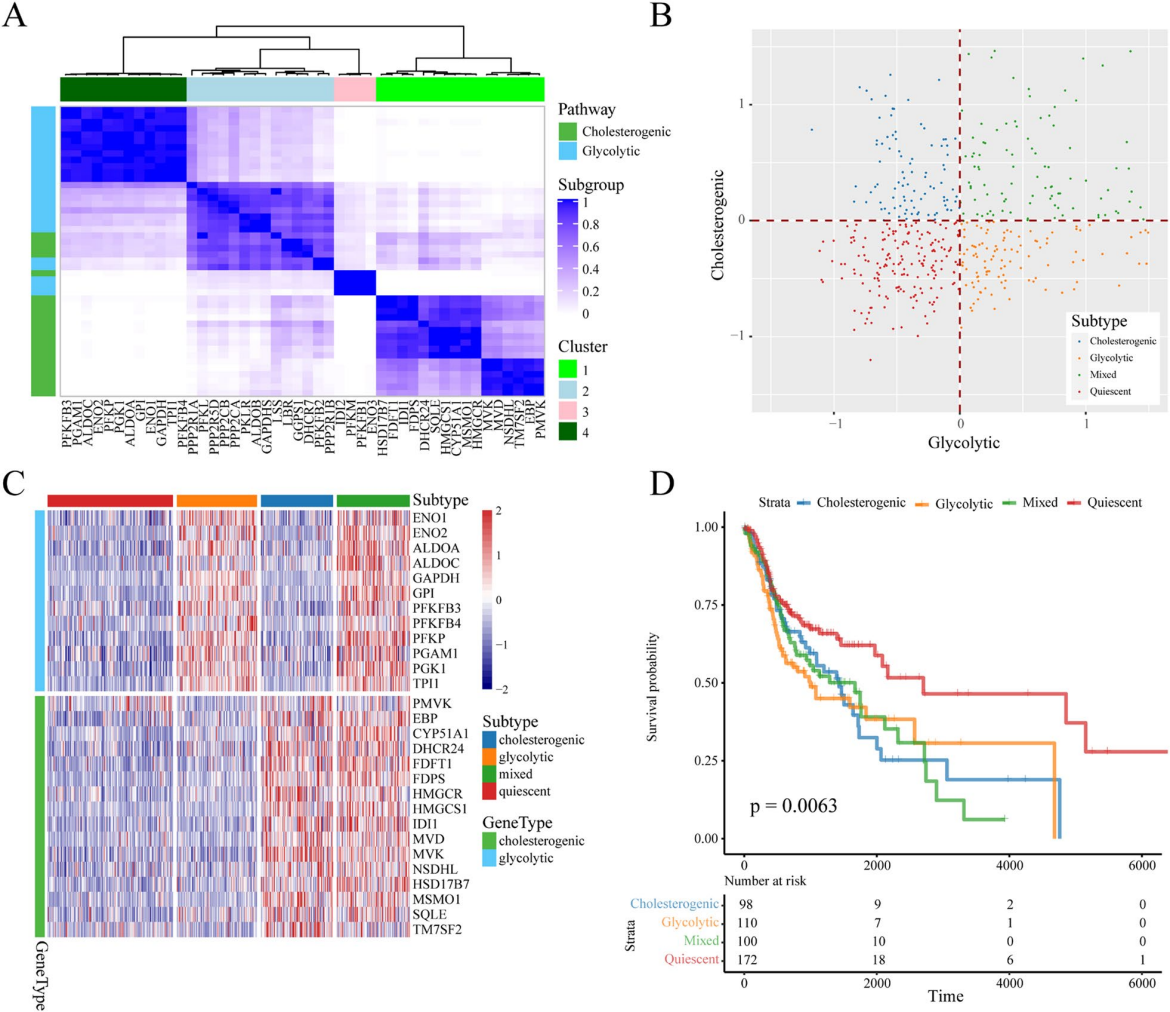
Stratification of TCGA HNSCC samples based on the expression of glycolytic and cholesterogenic genes. **A** Heatmap depicting consensus clustering solution (k = 4) for glycolytic and cholesterogenic genes in TCGA HNSCC samples. **B** Scatter plot showing median expression levels of coexpressed glycolytic (x-axis) and cholesterogenic (y-axis) genes in each HNSCC sample. Metabolic subgroups were assigned on the basis of the relative expression levels of glycolytic and cholesterogenic genes. **C** Heatmap depicting the expression levels of coexpressed glycolytic and cholesterogenic genes across each subgroup. **D** Kaplan–Meier survival curves of patients in four metabolic subtypes in TCGA. Log-rank test *P* values are shown

To reveal the metabolic heterogeneity of HNSCC based on the relative gene expression levels of glycolytic and cholesterol synthesis pathways, we downloaded RNA-seq data from HNSCC samples in TCGA. We obtained a dataset containing 481 samples and filtered out samples with low (< 30%) tumor cell content. Genes belonging to the molecular signature database gene sets “glycolysis” (n = 29) and “cholesterol biosynthesis” (n = 24) were identified as glycolytic and cholesterogenic genes for further analysis. We utilized a consensus clustering approach to identify two group clusters based on genes coregulated in metabolic pathways in HNSCC. According to the consensus cluster analysis, these genes in the glycolysis (n = 12) and cholesterol biosynthesis (n = 16) pathways were used for the HNSCC metabolic subtype (Fig. 1A).

Expression levels of both glycolysis and cholesterogenic genes as a profile in each sample were calculated and assigned to four metabolic subtypes in HNSCC, including the glycolysis, cholesterogenic, mixed and quiescent subtypes. The details are as follows. The glycolytic phenotype was characterized by remarkable upregulation of glycolytic genes and downregulation of cholesterogenic genes. The cholesterol phenotype was characterized by the relative upregulation of cholesterogenic genes and downregulation of glycolytic genes. The mixed phenotype was characterized by the combined upregulation of each major category. The quiescent phenotype was characterized by the combined downregulation of each major category (Fig. 1B). The expression levels of genes involved in glycolysis and cholesterogenesis across all metabolic subtypes in HNSCC are shown in Fig. 1C. The quiescent subtype made up the largest portion of HNSCC samples (172/481; 35.76%), followed by the glycolytic subtype (110/481; 22.87%), mixed subtype (100/481; 20.79%) and cholesterogenic subtype (99/481; 20.58%). We further performed survival analysis, since survival represents a key clinical index of tumor aggressiveness, and overall survival outcome were significantly different among the four HNSCC subtypes. The other three subtypes had significantly worse outcome than the quiescent subtype (Fig. 1D).

**Fig. 2:**
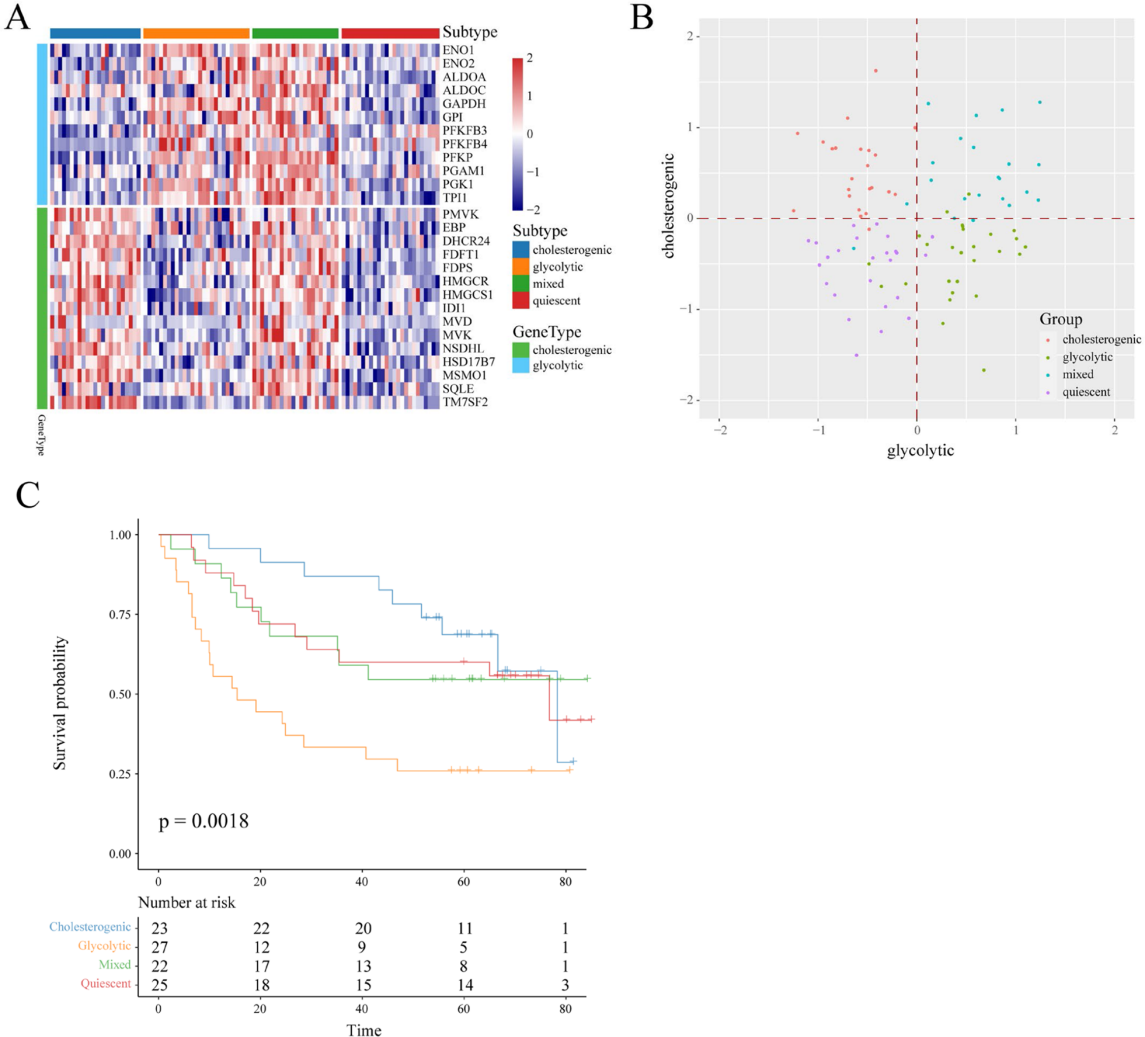
Stratification of GEO-HNSCC samples based on the expression of glycolytic and cholesterogenic genes. **A** Heatmap depicting the expression levels of coexpressed glycolytic and cholesterogenic genes across each subgroup. **B** Scatter plot showing median expression levels of coexpressed glycolytic (x-axis) and cholesterogenic (y-axis) genes in each HNSCC sample. Metabolic subgroups were assigned on the basis of the relative expression levels of glycolytic and cholesterogenic genes. **C** Kaplan–Meier survival curves of patients with four metabolic subtypes in GEO. Log-rank test *P* values are shown

To confirm the robustness of our classification model, RNA-seq data and metadata of 97 HNSCC samples from GEO (GSE41613) were applied for external validation of the gene signature. These genes in the glycolysis (n = 12) and cholesterol biosynthesis (n = 15) pathways were used for metabolic subtype according to the formula above (Fig. 2A, B). In 97 patients with HNSCC, glycolysis subtype, cholesterogenic subtype, quiescent subtype and mixed subtype cases accounted for 27.84% (27/97), 23.71% (23/97), 25.77% (25/97) and 22.68% (22/97), respectively. There was no statistically significant difference in the proportion of the four subtypes. Consistent with previous results, the quiescent subtype had better survival than the glycolysis subtype in the GEO validation dataset (Fig. 2C).

These findings indicated that multiple metabolic phenotypes were established in HNSCC and were involved in glycolysis or cholesterogenic biosynthesis. Compared with tumors of the quiescent subtype, we reasoned that tumors with a higher glycolysis subtype or higher cholesterogenic subtype are much more aggressive in HNSCC.

### Clinical relevance and molecular mechanism of the four metabolic subtypes in HNSCC

**Fig. 3:**
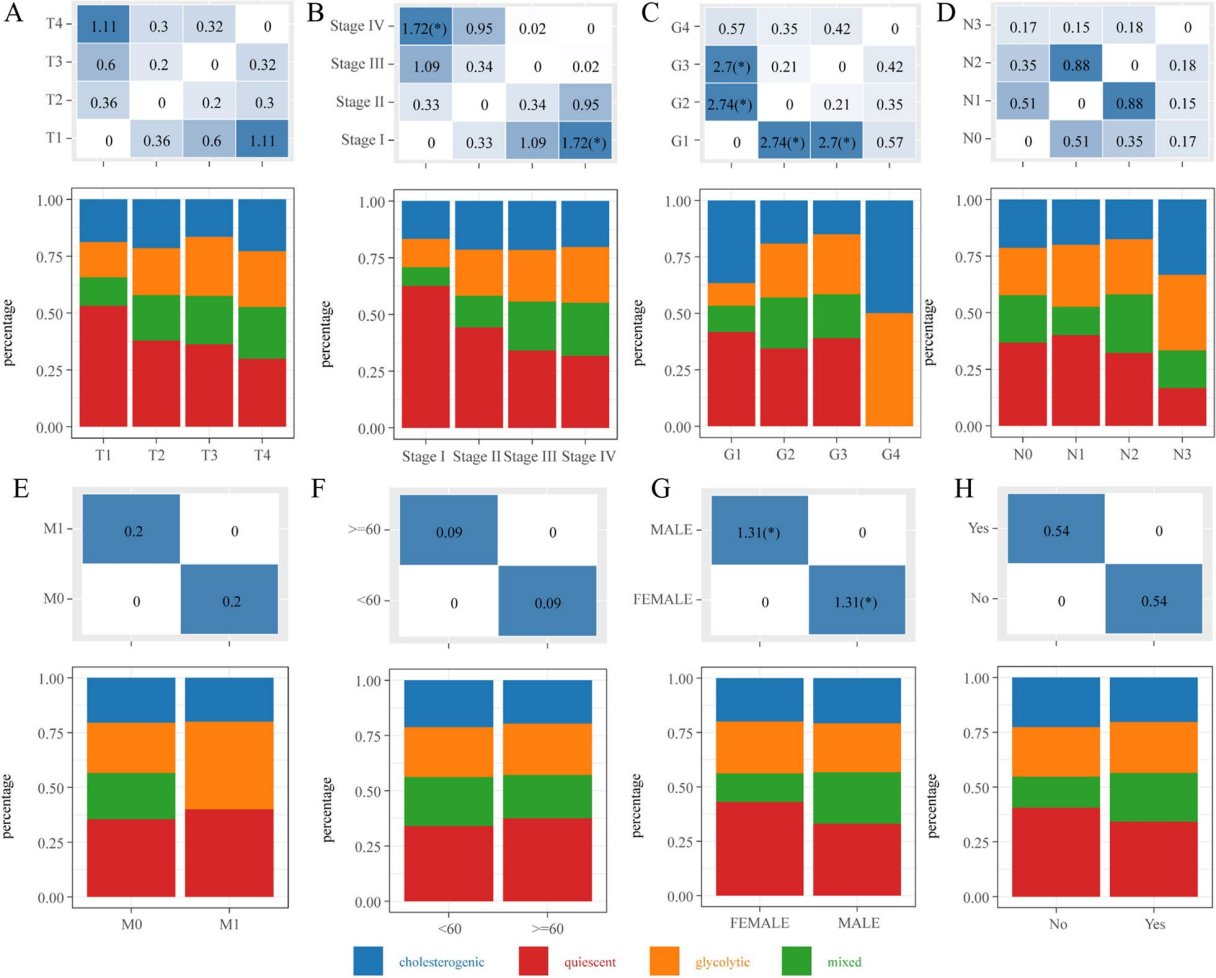
Factor analysis of four HNSCC subtypes based on clinical characteristics: (**A**) T stage, (**B**) clinical stage, (**C**) G stage, (**D**) N stage, (**E**) M stage, (**F**) age, (**G**) gender, (**H**) smoking history distribution in the four HNSCC subtypes

To construct a clinical matrix that predicts the prognosis of HNSCC patients among the four subtypes, clinicopathological factors, including tumor (T), node (N), metastasis (M), clinical stage, grade (G), age, gender and smoking history, were analyzed in TCGA HNSCC datasets. As illustrated in Fig. 3A, B, we observed that most quiescent subtypes were enriched in T1 stage and Stage I. Our results also showed that the G2-G4 stages consisted of more glycolysis subtype cases than the G1 stage (Fig. 3C). We found no difference in N, M, patient age or smoking history (Fig. 3D–H). Interestingly, there was no quiescent subtype in the G4 stage of HNSCC (Fig. 3C), suggesting metabolic alterations in HNSCC progression.

**Fig. 4:**
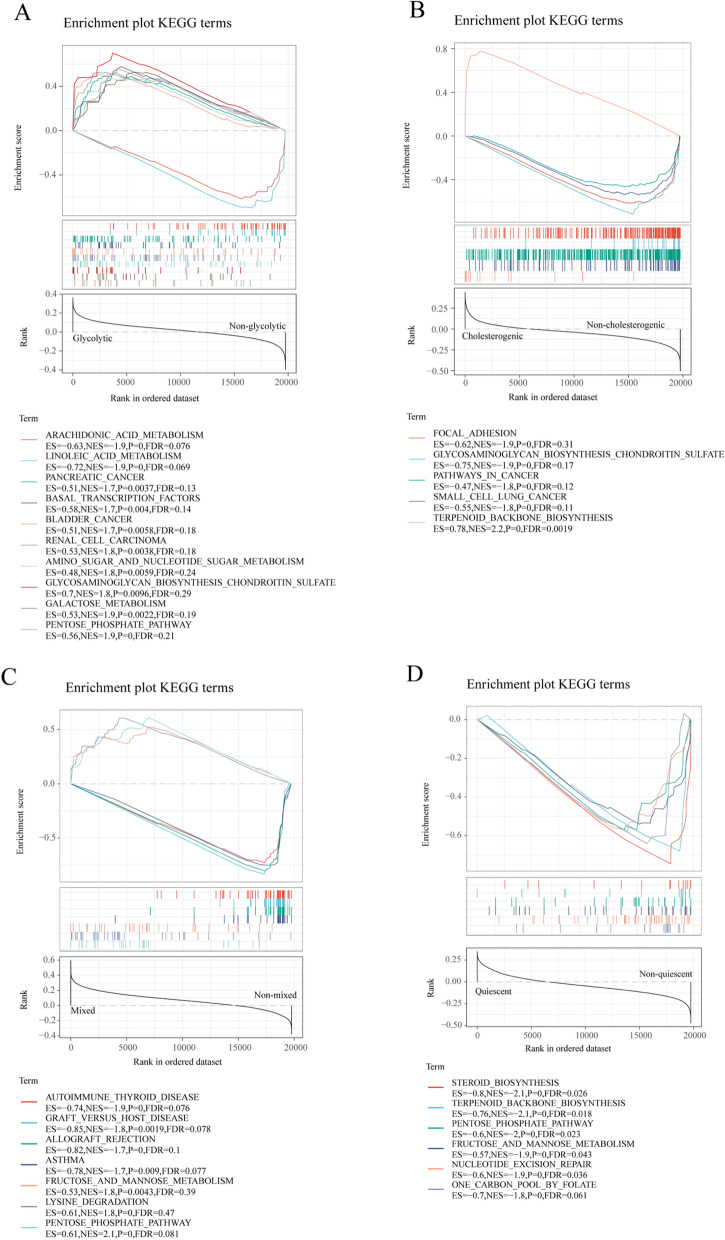
Metabolic subtype signature-associated biological signaling pathways by GSEA in TCGA-HNSCC samples. KEGG enrichment analysis results of genes: (**A**) in the glycolytic subtyp,. (**B**) in the cholesterogenic subtype, (**C**) in the mixed subtype, (**D**) in the quiescent subtype. Colors indicate the enriched signaling pathway

To investigate the underlying molecular mechanisms of the four metabolic subtypes, we conducted GSEA of HNSCC samples. The cholesterogenic and glycolysis subtypes were mainly involved in pathways in cancer (Fig. 4A, B), whereas the mixed subtype was enriched in pathways in immune disease (Fig. 4C). Importantly, the quiescent subtype mainly focused on DNA repair, and the glucocorticoid synthesis pathway indicated that the quiescent subtype had a better prognosis than the other three subtypes (Fig. 4D). In short, we proposed that metabolic alteration was associated with the worse prognostic outcome.

### Molecular subtypes identified by immunohistochemical markers

**Fig. 5:**
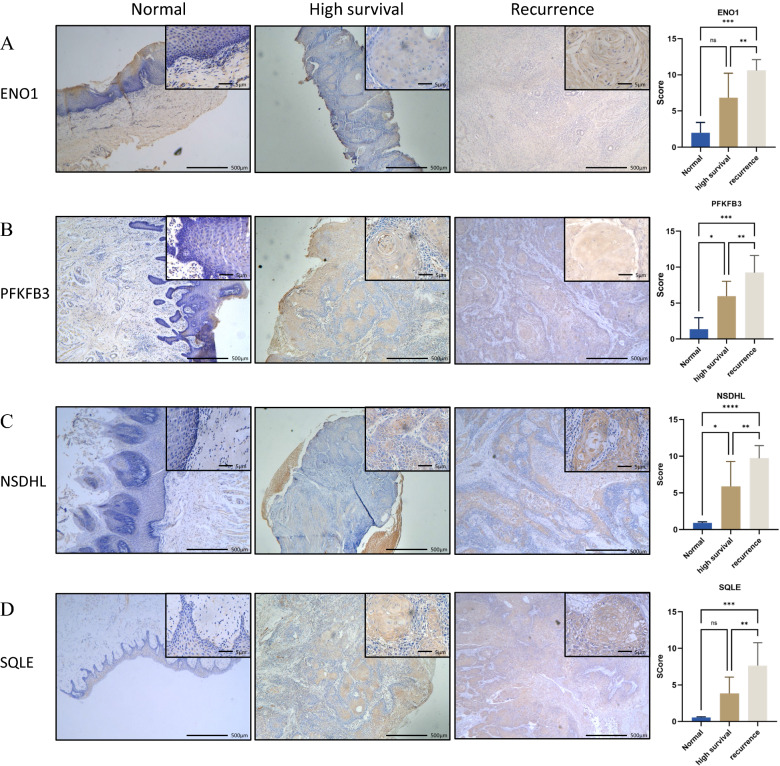
ENO1, PFKFB3, NSDHL and SQLE expression in HNSCC. **A** The typical staining and IHC score of ENO1 in normal tissues and HNSCC tissues from recurrent HNSCC patients (recurrence group) and first diagnosed HNSCC patients surviving at least 5 years (high survival group). **B** The typical staining and IHC score of PFKFB3 in in normal tissues and HNSCC tissues from recurrent HNSCC patients (recurrence group) and first diagnosed HNSCC patients surviving at least 5 years (high survival group). **C** The typical staining and IHC score of NSDHL in in normal tissues and HNSCC tissues from recurrent HNSCC patients (recurrence group) and first diagnosed HNSCC patients surviving at least 5 years (high survival group). **D** The typical staining and IHC score of SQLE in in normal tissues and HNSCC tissues from recurrent HNSCC patients (recurrence group) and first diagnosed HNSCC patients surviving at least 5 years (high survival group). Data were shown as mean ± SD (*P < 0.05, **P < 0.01, ***P < 0.001, ****P < 0.0001)

Expression levels of several selected glycolysis and cholesterogenic proteins corresponding to the top genes were examined to further characterize the different subtypes from tumor tissue with first diagnosed HNSCC patients surviving at least 5 years and recurrent HNSCC patients. These proteins include Enolase 1 (ENO1), Phosphofructo-2-kinase/fructose-2,6-biphosphatase 3 (PFKFB3), NADP steroid dehydrogenase like (NSDHL) and squalene epoxidase (SQLE) and we analyzed these protein expression levels by IHC. The results showed that ENO1, PFKFB3, NSDHL and SQLE were primarily detected in the in the cytoplasm of cancer cells in HNSCC tissues and the expression of these protein in recurrent HNSCC was significantly higher than that in normal tissues (Fig. 5A–D). The expression of PFKFB3 and NSDHL in recurrent HNSCC was significantly higher than that in the normal tissue (Fig. 5B, C). These data suggested that abnormal metabolism-related protein expression was founded to be HNSCC patients with poor prognosis. Furthermore, we observed that ENO1, PFKFB3, NSDHL and SQLE were highly expressed in recurrent HNSCC tumor compared with first diagnosed HNSCC patients surviving at least 5 years (Fig. 5A–D). Together, these findings suggested that tumors with a higher glycolysis or higher cholesterogenic proteins are worse survival in HNSCC.

### Association of the four metabolic subtypes in HNSCC with featured genomic alterations and the known HNSCC subtypes

**Fig. 6:**
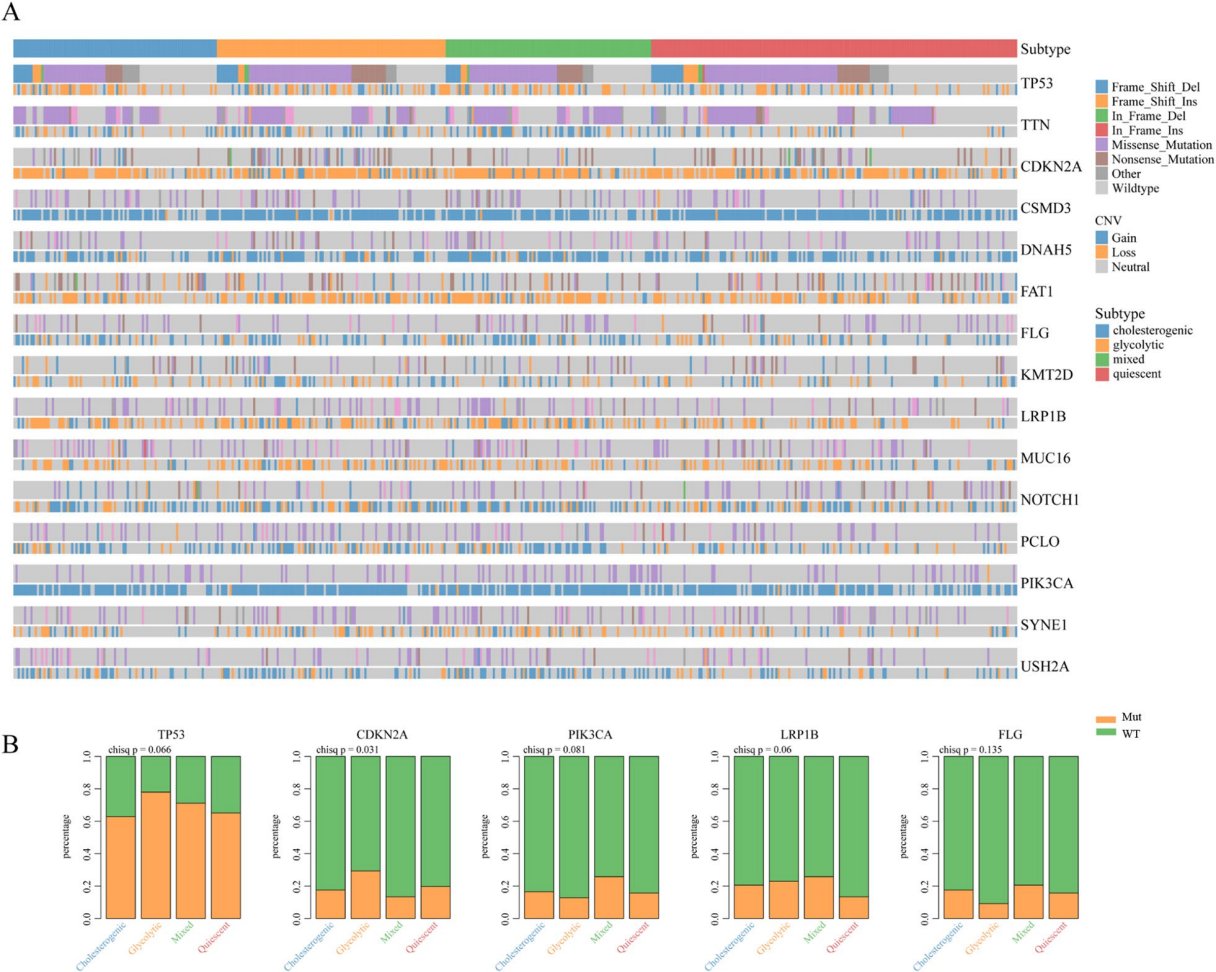
Mutational analysis in the four metabolic subtypes of HNSCC. **A** Somatic landscape depicting the distribution of somatic mutation (SNV/indel) and copy number variation (CNV) events affecting frequently mutated genes in the four metabolic subtypes. **B** Differences in the percentages of TP53, CDKN2A, PIK3CA, LRP1B and FLG mutations and wild-type genes among the four subtypes of HNSCC

Genomic alterations, such as oncogenic mutant TP53 or CDKN2A, could drive metabolic reprogramming in cancers, including HNSCC [35, 36]. We next used RNAseq data from TCGA HNSCC datasets to examine the frequency of insertion-deletion mutations (INDELs), single nucleotide variations (SNVs) and copy number variations (CNVs) across the metabolic subtypes (Additional file 1: Figure S2). The study indicated that TP53, CDKN2A, PIK3CA, LRP1B and FLG were the most frequently mutated genes among the four metabolic subtypes in HNSCC (Fig. 6A and Additional file 1: Figure S2). Of note, we observed that among the four subtypes, TP53 has the highest mutation frequency. The CDKN2A mutation frequency has the most significant differences amongst the four subtypes, whereas PIK3CA, LRP1B and FLG had marginally significant mutation frequencies (Fig. 6B). Overall, this result suggested that alterations in oncogenes might influence the metabolic reprogramming of HNSCC.

**Fig. 7:**
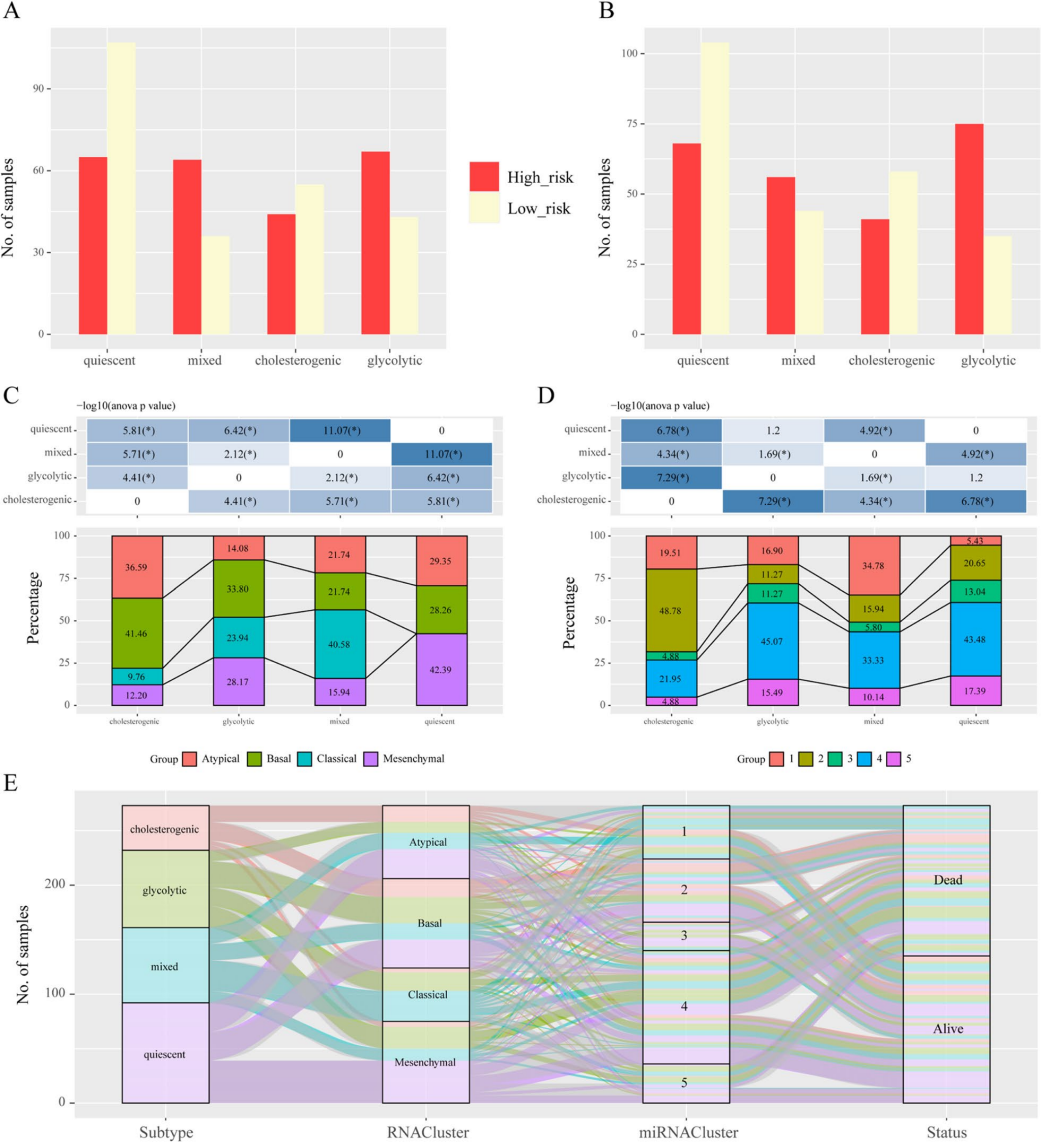
Relationships between metabolic subtypes and known HNSCC subtypes. **A**, **B** Bar plots illustrating the proportion of published HNSCC expression subtypes across each metabolic subgroup based on patient prognosis. **C** Factor analysis of four HNSCC subtypes based on mRNA-based subtyping. **D** Factor analysis of four HNSCC subtypes based on miRNA-based subtyping. **E** Sankey diagram showing overlay of the metabolic profiles with HNSCC expression subtypes based on mRNA-based subtyping by Chung and miRNA-based subtyping by Walter, as well as patient survival

Previous studies have reported a gene expression signature associated with survival time in patients with HNSCC [37]. According to the prognosis models of Zhang [30] and Zhou [31], we determined the risk score of HNSCC prognosis and investigated their overlap with the metabolic subtype in TCGA HNSCC datasets. The quiescent subtype contained the highest frequency of low-risk cases, and the cholesterogenic subtype was also mainly composed of low-risk samples. In contrast, glycolysis and the mixed subtype predominantly consisted of the high-risk prognosis group (Fig. 7A, B).

Moreover, Chung and Walter et al. identified molecular subtypes of HNSCC based on gene expression, termed basal, mesenchymal, classical and atypical [38–40]. The tumor subtype with the worst outcome was the classical classification [39]. Similarly, the basal and mesenchymal subtypes were also associated with poor outcome [38–40]. To validate our subtypes, we investigated the subtypes that corresponded to the Chung and Walter classification [40]. As shown in Fig. 7C, most of the basal subtypes (33.8% and 41.46%, respectively) of HNSCC were enriched in the glycolysis and cholesterogenic subtypes. The classical subtype (40.58%) was enriched in the mixed subtype, but the mesenchymal subtype (42.39%) was enriched in the quiescent subtype. Likewise, we assessed the overlaps between our metabolic subtypes and the miRNA classification in HNSCC [39]. The Group 4 subtype was related to poor outcome. Consistent with the glycolytic subtype conferring the worse prognosis as described above, the majority of these samples were part of the Group 4 subtype (Fig. 7D). Finally, a Sankey diagram for the association between the above three classifications and patient survival was generated (Fig. 7E). Taken together, these data implied that metabolic alteration may influence the prognosis of the known HNSCC subtypes and that glycolysis biosynthesis might be a potential target for HNSCC therapy.

### Relevance of cholesterogenic and glycolytic gene clusters in other cancers

**Fig. 8:**
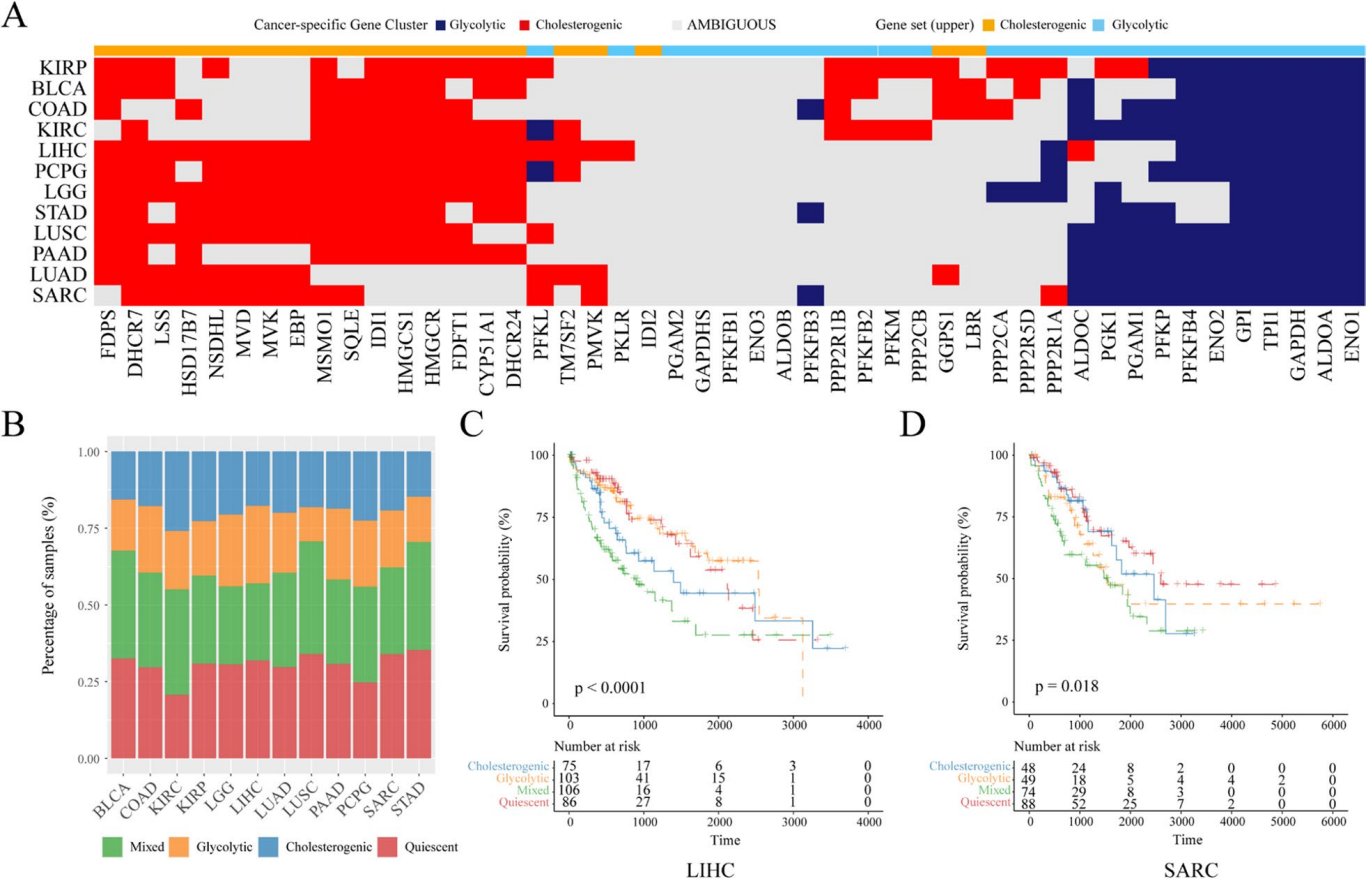
Glycolytic and cholesterogenic gene profiling of other cancer types. **A** Heatmap depicting which glycolytic and cholesterogenic genes were robustly coexpressed when consensus clustering was applied to each individual cancer type. **B** The distributions of different tumor types in four metabolic subtypes. **C** Kaplan–Meier survival analysis curves showing differences in overall survival across metabolic subgroups in LIHC. **D** Kaplan–Meier survival analysis curves showing differences in overall survival across metabolic subgroups in SARC. Log-rank test *P* values are shown

Diverse cancers show distinct metabolic signatures resulting from enzyme expression in specific organs and mutational landscapes, which may affect outcome. To determine the relevance of the expression levels of cholesterogenic and glycolytic genes at additional organ sites, consensus clustering was repeated to analyze the expression levels of cholesterogenic and glycolytic genes among the 12 types of TCGA cancers. Most genes showed coexpression in the majority of cancers, and only a few of them had coexpressed genes involved in the cholesterogenic and glycolytic pathways. Furthermore, these data indicated that some genes contributed to metabolic programs in every cancer type specific to the cell type (Fig. 8A, B). The candidate relationships of specific metabolic genes with the clinicopathological characteristics of different cancers were also examined. The difference in survival rates was significant across the four metabolic subtypes in liver hepatocellular carcinoma (LIHC) *(P* = 0.0001) (Fig. 8C) and sarcoma (SARC) (*P* = 0.018) (Fig. 8D). The OS rate of LIHC markedly decreased in the mixed subgroup compared with the cholesterogenic subgroup. For SARC, the glycolytic and mixed subgroups showed poor prognostic outcome relative to those of the quiescent and cholesterogenic subgroups. Therefore, this model shed new light on the molecular characteristics of different cancers and illustrated tumor-specific gene expression in different metabolic subtypes.

## Discussion

As several targeted therapies have been approved for HNSCC treatment and many more are in progress, the identification of predictive biomarkers to establish therapeutic guidelines is a major research priority. Specifically, dysregulated metabolism was previously reported to be associated with clinical outcome in diverse cancers [7, 8]. Several studies also conducted metabolic subtype based on metabolic dysregulation and heterogeneity of tumors, suggesting that metabolic profile studies may be a novel approach to identify tumor-specific targets for diagnosis and therapy [25, 27, 41]. However, to date, no research has defined the metabolic classification of HNSCC. Here, we successfully established four distinct subtypes of HNSCC, the quiescent, glycolytic, cholesterogenic and mixed subtypes, which affect tumor progression and patient survival.

Furthermore, we found that the metabolic subtype of HNSCC was linked to clinicopathological features and, in particular, to clinical stage and grade. The quiescent subtype was mainly enriched in stage I and G1, suggesting a better outcome. Previous studies have shown that lactate levels are not correlated with presenting T stage or N stage [42]. In accordance with previous studies, our results showed that T stage and N stage were not significantly different in the distribution of the metabolic subgroups. Our study demonstrated that metabolomic profiling could be potentially useful for prognosis.

Glycolytic metabolism is a common event in tumorigenesis, as indicated by the dramatic increase in glucose uptake [22]. The finding that tumors with increased glycolytic properties were related to the shortest overall survival confirms the role of glycolysis in tumor aggressiveness in HNSCC [43]. Chen et al. found that six glycolysis-based genes were identified and can be used as prognostic markers for patients with HNSCC. In addition, lipid metabolic reprogramming is one hallmark of cancer. Cholesterol plays a key role in pathways governing carcinogenesis and malignant progression. High expression levels of cholesterogenic genes were associated with human HNSCC development and supported poor prognosis [44–47]. Emerging evidence supports these observations. ENO-1 acts as a glycolytic enzyme and promotes invasion and metastasis formation in various cancers [48–50]. PFKFB3 is an essential glycolysis-activating enzyme, and its powerful kinase activity can increase glycolysis flux and was involved in the aggressive features of multiple malignances and correlates with poor survival [51–53]. SQLE, a rate-limiting enzyme in the cholesterol synthesis, aggravates malignant progression in multiple cancers [54, 55]. NSDHL is also a cholesterol synthesis, which related to cancer growth and the signaling of proto-oncogenes [56]. Our study demonstrated that the other three subtypes including glycolytic, cholesterogenic and mixed subtypes, had significantly worse outcome than the quiescent subtype, which was characterized by abnormal expression of metabolism-related genes. We further validated the metabolism-related subtypes based on features of ENO1, PFKFB3, NSDHL and SQLE expression in HNSCC. Our data showed that both ENO1, PFKFB3, NSDHL and SQLE have high expression levels in clinical HNSCC samples, and all of them presented higher expression in patient with recurrence tumor. However, these proteins were almost no expression in normal tissue.

Metabolic reprogramming can be largely viewed as a consequence of oncogenic driver events [57]. With the aim of confirming the molecular drivers of distinct metabolic subtypes in HNSCC, we observed that TP53 has the highest mutation frequency and the CDKN2A mutation frequency has the most significant differences amongst these four subtypes. Previous studies in multiple cancers have identified a role for oncogenes, including CDKN2A, also called p16, as regulators of a range of tumor cell processes directly and indirectly contributing to the regulation of many different metabolic pathways. CDNK2A participates in metabolism control by regulating glucose transporter 1 (GLUT1) expression, which mediates glucose uptake in pancreatic cancer [36, 58]. Also, mutant p16 can induce oxidation of NADH and maintain glycolysis by generating NAD ^+^ , a substrate for GAPDH-mediated glycolytic reaction, thereby promoting pancreatic ductal adenocarcinoma development [59]. In addition, TP53 mutation have been extensively linked to the promotion of glycolysis through sustaining high fuel oxidation and ATP production in cancer [60, 61]. The current study indicates that different metabolic subtypes with distinct molecular profiles will provide potential subtype-specific therapeutic targets.

There is significant molecular heterogeneity in HNSCC leading to distinct tumor subtypes based on immune checkpoints, epigenetic modification and genetic alterations [1, 38–40]. Analysis of available expression datasets revealed that the four metabolic subtypes are reproducible in HNSCC and have a few overlaps with other molecular subtypes found in HNSCC. Notably, a clear pattern of correlation was observed in which the glycolysis and cholesterogenic subtypes of HNSCC corresponded to the HNSCC basal subtype, which is known to be associated with worse clinical outcome. In addition, the mesenchymal subtype is often described as a poor outcome. Our results showed that the quiescent subtype was enriched in the mesenchymal subtype. This implied the heterogeneity in HNSCC. Understanding the metabolic profiles of different molecular subtypes is important in heterogeneous diseases such as HNSCC, which contributes to a growing interest in translating this information into clinical practice for outcome prognostication and the development of personalized treatments based on each tumor's unique molecular signature.

The metabolic expression subtypes defined here have potential clinical implications. We showed that the extensive correlations of metabolic subtypes with prognosis in HNSCC, suggesting that the subtypes reflect essential aspects of tumor aggressiveness. Furthermore, current strategies for considering the effect of metabolism on therapy focus on functionally important metabolic gene that show cancer-specific somatic or expression changes. Overall, the results here support the potential utility of metabolic subtypes as prognosis and guideline for therapy. However, there are still some limitations in our study. First, the data we studied were from public databases rather than our database, the four metabolism subtypes should be further identified in large patient samples. Second, the mechanisms underlying metabolic regulation and HNSCC metabolic subtypes of several metabolism-related genes included in the mutation data needed to be explored.

A vital outcome of the work in cancer metabolism is our ability to translate these findings into personalized therapeutic approaches. Several metabolic inhibitors already in pre-clinical and clinical investigation. For example, 2-DG, antiglycolytic agent, acts as a competitive inhibitor of the glycolytic enzyme hexokinase. And there is currently a clinical trial in advanced cancer (Targeting EMT in Cancer with Repurposed Metabolic Inhibitors). ABT-510, which inhibits FA and LDL protein uptake, were both found to be highly effective for secondary lymph node metastatic tumor development and has been tested in clinical trials [62]. Although these inhibitors have limited therapeutic effect in many types of cancers, it may be combined with other therapeutic agents to exhibit a synergistic anticancer effect. Here, the correlation of gene expression heterogeneity along the glycolysis and cholesterol and prognosis subtypes of HNSCC have shown that subtype-specific therapeutic strategies targeting unique metabolic vulnerabilities, together with conventional antineoplastics. One treatment strategy is the glycolytic subtype may be targeted by combining inhibition of glycolysis with CDKN2A or TP53. On the other hand, targeting both cholesterol and CDKN2A or TP53 in the cholesterogenic subtype may be an efficient therapy. Therefore, we need further explore the key metabolic gene and mutation among the four subtypes.

## Conclusions

In summary, the four metabolic subtypes were successfully determined in HNSCC. Compared to the quiescent subtype, glycolytic, cholesterogenic and mixed subtypes had significantly worse outcome, which might offer guidelines for developing a novel treatment strategy for HNSCC.

## Supplementary Information

**Additional file 1: Figure S1.** The consensus clustering of HNSCC samples classification. **A**–**D** The color‐coded heatmap corresponding to the consensus matrix for k = 2,3,4,5 obtained by applying consensus clustering. The color gradients were from 0 to 1, representing the degree of consensus, with white corresponding to 0 and dark blue to 1. **E**–**F** Delta area curve of consensus clustering, indicating the relative change in area under the cumulative distribution function (CDF) curve for 2 each category number k compared with k–1. The horizontal axis represents the category number k and the vertical axis represents the relative change in area under CDF curve. **Figure S2.** This maftools plot showing most mutated genes, SNV class, and variant classification distributions in HNSCC. **Table S1.** Clinicopathological characteristics of patients with head and neck squamous cell carcinoma (N = 22).

## Data Availability

The original contributions presented in the study are included in the article/Supplementary Material.The datasets presented in this study can be found in online repositories.
