# Supplementary material for: Prognosis-related molecular subtyping in head and neck squamous cell carcinoma patients based on glycolytic/cholesterogenic gene data
Source: Cancer Cell Int. 2023 Feb 25;23:37. doi: 10.1186/s12935-023-02880-3 (PMC9960414; doi:10.1186/s12935-023-02880-3)
Supplement: Supplementary file 1 — Additional file 1: Figure S1. The consensus clustering of HNSCC samples classification. A–D The color‐coded heatmap corresponding to the consensus matrix for k = 2,3,4,5 obtained by applying consensus clustering. The color gradients were from 0 to 1, representing the degree of consensus, with white corresponding to 0 and dark blue to 1. E–F Delta area curve of consensus clustering, indicating the relative change in area under the cumulative distribution function (CDF) curve for 2 each category number k compared with k–1. The horizontal axis represents the category number k and the vertical axis represents the relative change in area under CDF curve. Figure S2. This maftools plot showing most mutated genes, SNV class, and variant classification distributions in HNSCC. Table S1. Clinicopathological characteristics of patients with head and neck squamous cell carcinoma (N = 22). [file 12935_2023_2880_MOESM1_ESM.pdf]

Supplementary Material

1 Supplementary Figures

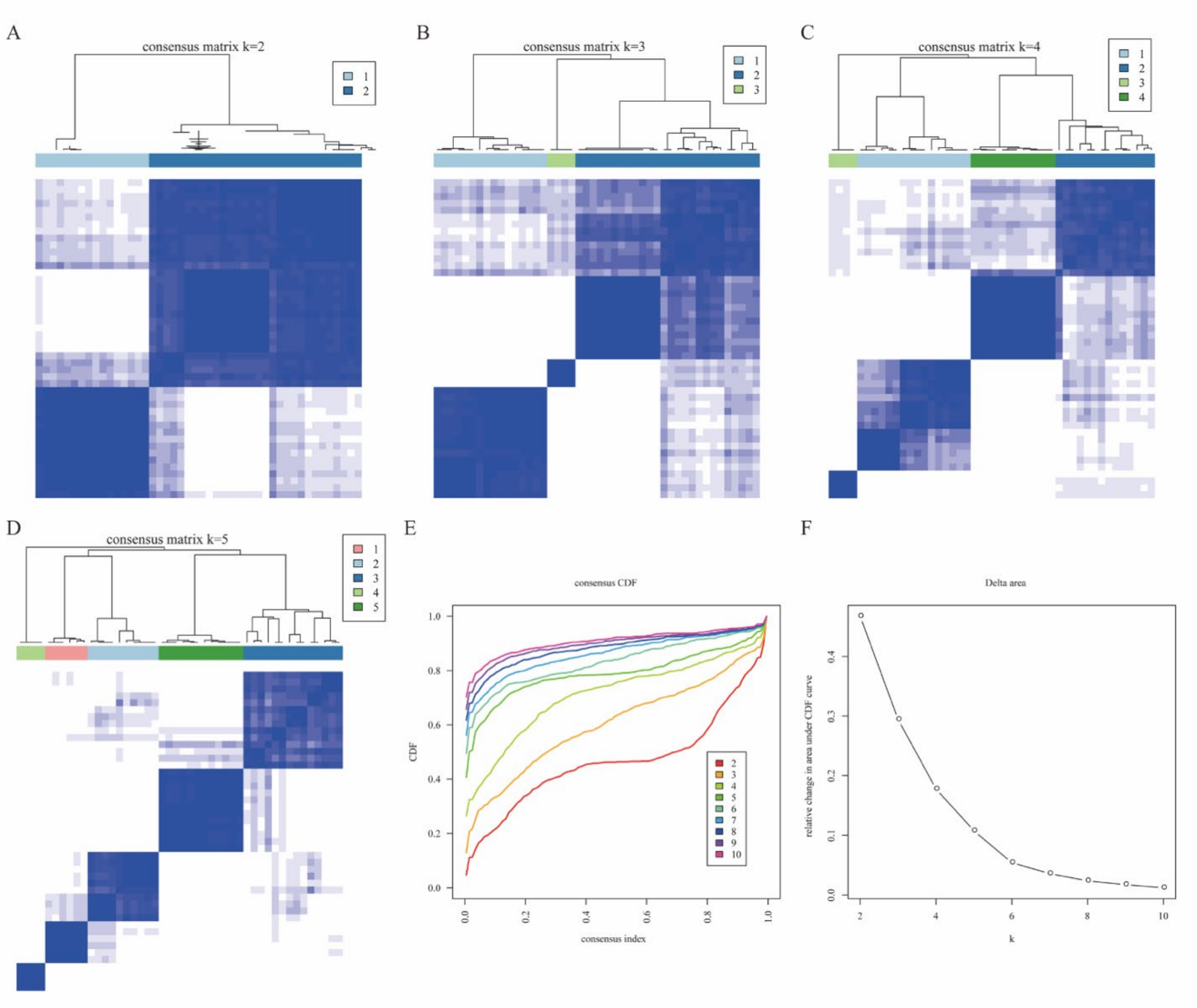

Figure S1. The consensus clustering of HNSCC samples classification. **(A-D)** The color-coded heatmap corresponding to the consensus matrix for k = 2,3,4,5 obtained by applying consensus clustering. The color gradients were from 0 to 1, representing the degree of consensus, with white corresponding to 0 and dark blue to 1. **(E-F)** Delta area curve of consensus clustering, indicating the relative change in area under the cumulative distribution function (CDF) curve for

each category number k compared with k–1. The horizontal axis represents the category number k and the vertical axis represents the relative change in area under CDF curve.

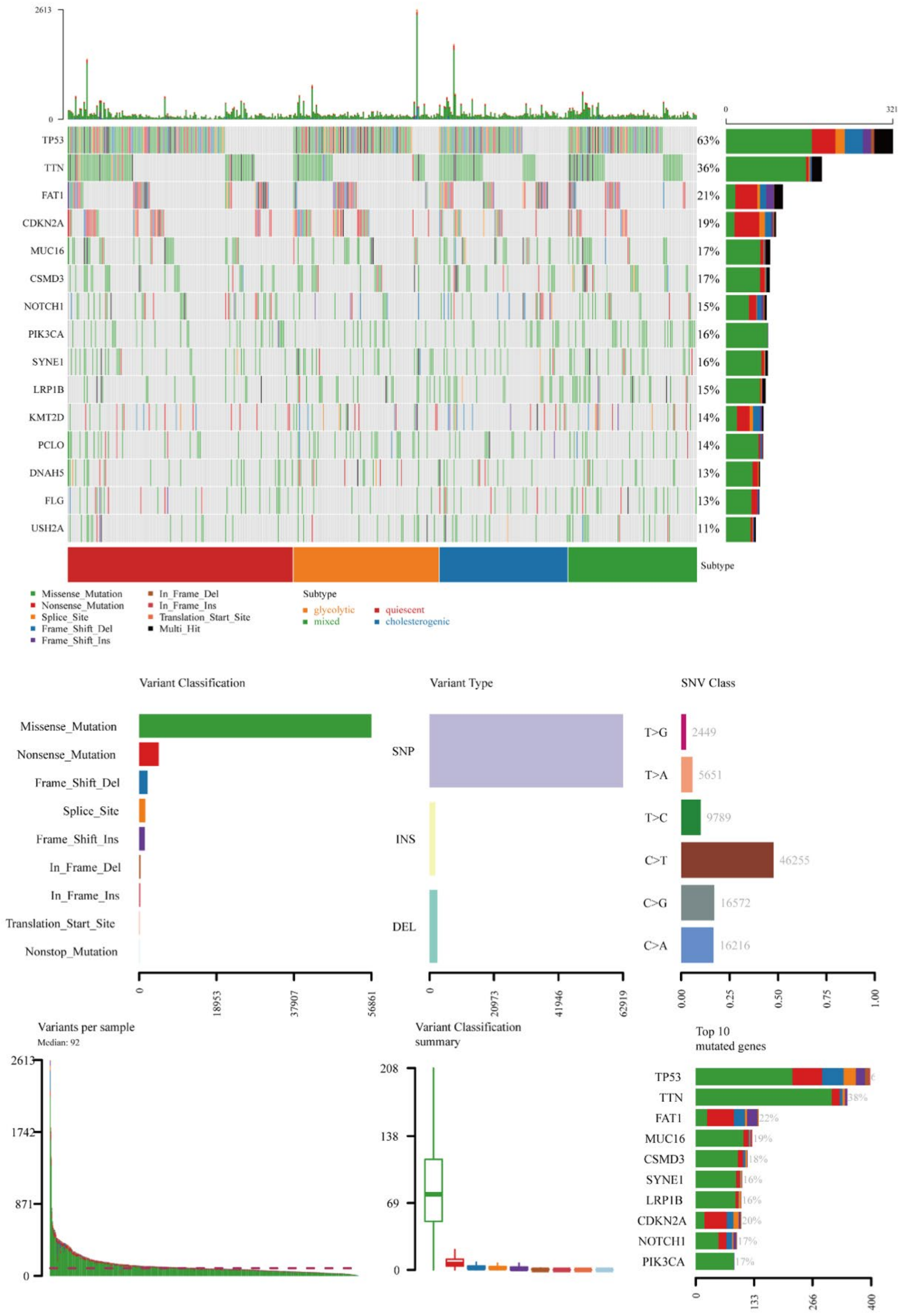

Figure S2. This maftools plot showing most mutated genes, SNV class, and variant classification distributions in HNSCC.

2 Supplementary Table

Table S1. Clinicopathological characteristics of patients with head and neck squamous cell carcinoma (N = 22)

Characteristics	n
**Gender**	
male	17
female	5
**Age**	
≤60	11
>60	11
**T stage**	
T1-T2	13
T3-T4	9
**N stage**	
N0	4
N1-N3	18
**Location**	
Tongue	10
Buccal	6

Oropharynx	2
Nasopharynx	2
Mouth floor	1
Gingiva	1
